# Rifaximin Ameliorates Non-alcoholic Steatohepatitis in Mice Through Regulating gut Microbiome-Related Bile Acids

**DOI:** 10.3389/fphar.2022.841132

**Published:** 2022-04-04

**Authors:** Jie Jian, Mei-Tong Nie, Baoyu Xiang, Hui Qian, Chuan Yin, Xin Zhang, Menghui Zhang, Xuan Zhu, Wei-Fen Xie

**Affiliations:** ^1^ Department of Gastroenterology, First Affiliated Hospital of Nanchang University, Nanchang, China; ^2^ Department of Gastroenterology, Shanghai East Hospital, Tongji University School of Medicine, Shanghai, China; ^3^ Shanghai Institute of Stem Cell Research and Clinical Translation, Shanghai, China; ^4^ State Key Laboratory of Microbial Metabolism, Joint International Research Laboratory of Metabolic and Developmental Sciences, and School of Life Sciences and Biotechnology, Shanghai Jiao Tong University, Shanghai, China; ^5^ Department of Gastroenterology, Changzheng Hospital, Naval Medical University, Shanghai, China

**Keywords:** rifaximin, non-alcoholic steatohepatitis, gut microbiome, bile acids, deoxycholic acid, farnesoid X receptor

## Abstract

Non-alcoholic steatohepatitis (NASH) is the progressive stage of non-alcoholic fatty liver disease (NAFLD). The non-absorbable antibiotic rifaximin has been used for treatment of irritable bowel syndrome, traveling diarrhea, and hepatic encephalopathy, but the efficacy of rifaximin in NASH patients remains controversial. This study investigated the effects and underlying mechanisms of rifaximin treatment in mice with methionine and choline deficient (MCD) diet-induced NASH. We found that rifaximin greatly ameliorated hepatic steatosis, lobular inflammation, and fibrogenesis in MCD-fed mice. Bacterial 16S rRNA sequencing revealed that the gut microbiome was significantly altered in MCD-fed mice. Rifaximin treatment enriched 13 amplicon sequence variants (ASVs) belonging to the groups *Muribaculaceae*, *Parabacteroides*, *Coriobacteriaceae_UCG-002*, *uncultured Oscillospiraceae*, *Dubosiella*, *Rikenellaceae_RC9_gut_group*, *Mucispirillum*, and *uncultured Desulfovibrionaceae*. However, rifaximin treatment also reduced seven ASVs in the groups *Aerococcus*, *Oscillospiraceae*, *uncultured Ruminococcaceae*, *Bilophila*, *Muribaculaceae*, *Helicobacter*, and *Alistipes* in MCD-fed mice. Bile acid-targeted metabolomic analysis indicated that the MCD diet resulted in accumulation of primary bile acids and deoxycholic acid (DCA) in the ileum. Rifaximin delivery reduced DCA levels in MCD-fed mice. Correlation analysis further showed that DCA levels were associated with differentially abundant ASVs modulated by rifaximin. In conclusion, rifaximin may ameliorate NASH by decreasing ileal DCA through alteration of the gut microbiome in MCD-fed mice. Rifaximin treatment may therefore be a promising approach for NASH therapy in humans.

## Introduction

Non-alcoholic fatty liver disease (NAFLD) is one of the most common chronic liver diseases worldwide. Approximately 20–30% of the generally Western population and 25% of Chinese people are estimated to suffer from NAFLD (Younossi et al., 2019; Lin et al., 2020). The early stage of NAFLD primarily manifests as hepatic steatosis with increased serum cholesterol and triglyceride levels. Non-alcoholic steatohepatitis (NASH), the progressive subtype of NAFLD, is characterized by liver steatosis, hepatocellular injury, inflammation, and different degrees of fibrosis (Ni et al., 2017; Friedman et al., 2018). NASH is a pathogenic factor for end-stage liver diseases such as cirrhosis and hepatocellular carcinoma (HCC) (Kim et al., 2018; Zhang et al., 2021). Although the high prevalence of NAFLD and related chronic liver diseases is a great public health concern, many clinical trials for NAFLD treatments have failed, and there is still no approved effective medicine to treat NASH (Haas et al., 2016; Francque, 2021; Francque et al., 2021).

It is well recognized that gut microbiome composition is essential for the progression of NAFLD. Bacterial fermentation metabolites, including short-chain fatty acids (SCFAs), succinate, ethanol, and bile acids, have been reported to affect the occurrence and progression of NAFLD (Gil-Gomez et al., 2021). Primary bile acids, are synthesized in the liver, followed by conjugation and transported to the intestine. Such primary bile acids include chenodeoxycholic acid (CDCA) and cholic acid (CA) in humans, and CA, muricholic acids (MCAs), and ursodeoxycholic acid (UDCA) in rodents (Wahlstrom et al., 2016). Intestinal bacteria convert primary bile acids into secondary bile acids, such as deoxycholic acid (DCA), tauroursodeoxycholic acid (TUDCA) and lithocholic acid (LCA) (Wahlstrom, et al., 2016). One of the most essential functions of these bile acids is to regulate farnesoid X receptor (FXR), which influences lipid metabolism (Fiorucci et al., 2021). A semisynthetic derivative of CDCA named obeticholic acid (OCA) is an agonist of FXR (Pellicciari et al., 2002). In Phase II and III trials, OCA reduced NASH-induced fibrosis (Fiorucci et al., 2019). A previous study revealed that the inhibition of intestinal FXR signaling by glycoursodeoxycholic acid (GUDCA) reduces the expression of fibroblast growth factor 15 (FGF15) and subsequently reverses lipid metabolic dysregulation in obese mice (Sun et al., 2018). Moreover, blocking bile acid transportation in the intestine was associated with decreased lipid accumulation in the liver of high fat diet (HFD)-fed mice (Rao et al., 2016; Arab et al., 2017). Thus, gut microbiome-related bile acid metabolic disorders may play important roles in NAFLD.

Rifaximin is a non-systemic antibiotic targeting a broad range of bacteria in the gastrointestinal tunnel with minimal absorption. It has been used clinically for the treatment of irritable bowel syndrome, traveling diarrhea and hepatic encephalopathy (Bass et al., 2010; Caraceni et al., 2021). Rifaximin is also used for the treatment of NASH in rats and humans, but the efficacy of this treatment remains controversial. A study by Cheng et al., in 2012 revealed that chronic exposure to rifaximin (6 months) led to hepatic steatosis in pregnane X receptor-humanized mice, demonstrating this is a potential side effect of rifaximin (Cheng et al., 2012). A recent report by Yukihisa et al. indicated that rifaximin combined with angiotensin-II receptor blocker attenuated choline-deficient/l-amino acid-defined (CDAA) diet-induced NASH fibrosis in rat (Fujinaga et al., 2020). Clinically, Gangarapu et al. found that short-term rifaximin treatment (1,200 mg/day) for 28 days decreased the level of serum endotoxemia and increased liver function in NASH patients (Gangarapu et al., 2015). Abdel-Razik et al. also reported that rifaximin administration (1,100 mg/day) for 6 months reduced NAFLD liver fat scores and improved serum endotoxemia, insulin resistance, and proinflammatory cytokine levels (Abdel-Razik et al., 2018). However, Cobbold et al. showed that a 6-week treatment with rifaximin (800 mg/day) had no beneficial effect on NASH (Cobbold et al., 2018). Therefore, the role of rifaximin in NASH treatment requires further clarification.

In the present study, we aimed to investigate the potential benefits of rifaximin in treating methionine and choline deficient (MCD) diet-induced NASH in mice (Zhu et al., 2019). We also analyzed the effect of rifaximin on the gut microbiome and bile acids profile. Our results provide new insights into potential clinical treatment of NAFLD by using rifaximin to alter the gut microbiome and associated metabolites.

## Methods and Materials

### Animals and Treatments

Male C57BL/6 wild-type littermates (6 weeks old, weighing nearly 20 g, obtained from the Shanghai Experimental Center of Chinese Academy of Sciences) were fed with an MCD diet (TP 3005M, Trophic Animal Feed High-tech Company, China) or standard methionine-and choline-sufficient (MCS) diet (TP 3005 GS, Trophic Animal Feed High-tech Company, China) ad libitum for 6 weeks. To evaluate the effects of rifaximin, a dose of 100 mg/kg/day rifaximin was administered by gavage to eight mice 2 weeks after beginning the MCD diet and continuing for 4 weeks. The control mice were gavage by saline. All mice were housed at the experimental animal center of Second Military Medical University in a specific pathogen-free environment at 24°C temperature under a 12/12 h light/dark cycle. At experimental end points, mice were euthanized and samples were harvested. Body and liver weight were recorded.

### Histological Studies

Mouse liver sections fixed with 10% formalin were embedded in paraffin, then sliced into 4 μm-thick sections and stained with hematoxylin and eosin (H&E) for histopathological examination. Two investigators blinded to the treatments independently assigned a Non-alcoholic Fatty Liver Disease Activity Score (NAS) for each sample. NAS is a composite score including steatosis, lobular inflammation, and cytological ballooning (Puri and Sanyal 2012). Steatosis was scored on a scale of 0–3 based on low to medium power evaluation of parenchymal involvement according to the following criteria: 0 (<5%), 1 (5–33%), 2 (33–66%), or 3 (>66%). Inflammation was scored on a scale of 0–3 by overall assessment of all inflammatory foci according to the following criteria: 0 (No foci), 1 (<2 foci per 20x optical field), 2 (2-4 foci per 20x optical field), or 3 (>4 foci per 20x optical field). Hepatocellular ballooning was scored on a scale of 0–2 based on evaluation of enlarged vacuolated cells proportion according to the following criteria: 0 (none), 1 (mild, few), or 2 (moderate, many) (Puri and Sanyal 2012). Oil Red O staining was performed on liver sections fixed in 4% paraformaldehyde or frozen samples embedded in optimal cutting temperature compound (OCT). Samples were cut with a cryostat into 8-μm sections to evaluate the degree of hepatocyte steatosis. Fibrosis was assessed in liver sections embedded in paraffin and stained with Sirius Red. The intensity of steatosis and fibrogenesis were measured based on the percentage of tissue area stained with Oil Red O staining or Sirius Red using the image analysis software IMAGE-PRO Plus 6.0 (Media Cybernetics, United States).

### Immunohistochemistry

Immunohistochemical staining was performed on the paraffin-embedded liver sections according to standard procedures. Liver sections were deparaffinized with 100% xylene, rehydrated with an ethanol gradient followed by 100% ethanol, 95% ethanol, 85% ethanol, and 75% ethanol, and immersed in 3% H2O2 to remove endogenous peroxidase. After antigen retrieval, slides were incubated with the primary antibodies α-SMA (ab5694, Abcam, Cambridge, United Kingdom) and Col1a1 (BA0325, Boster, Wuhan, China) overnight at 4°C. Samples were then incubated with horseradish peroxidase-linked immunoglobulin G secondary antibody (GK500710, Gene Tech, Shanghai, China) at room temperature for 1 h. Staining was performed using an Envision Detection Rabbit/Mouse Kit (GK500710, Gene Tech).

### Quantitative Real-Time PCR Analysis

Total RNA was extracted from liver and terminal ileum tissues using TRIzol Reagent (Invitrogen, Carlsbad, CA, United States). The 20 µl first-strand cDNA synthesis consists with 2 µg of total RNA resolved in RNase free water and retrotranscribed with 4 µl PrimeScript RT Master Mix (Takara, Kyoto, Japan). For quantitative PCR, cDNA was reverse-transcribed and then amplified with SYBR Green PCR Kit (Life Technologies, Carlsbad, CA, United States). PCR conditions were as follows: 95°C 10 min, then 40 cycles at 95°C for 30 s and 60°C for 1 min mRNA levels were normalized to those of Gapdh mRNA. Primers for Acta2, Col1a1, Cyp7a1, Cyp7b1, Cyp8b1, Cyp27a1, Fxr, Fgf15, and Shp are shown in Supplementary Table S1.

### Western Blot Analysis of Hepatic Proteins

Western blot was performed on liver protein extracts using RIPA buffer as previously described, including Srebp1, Pparγ, α-SMA, Col1a1, and Gapdh (Qian et al., 2015). Gapdh was used as the loading control for total protein. Primary antibodies are listed in Supplementary Table S2.

### Lipopolysaccharide Measurement

Serum LPS was quantified using a commercial enzyme-linked immunosorbent assay (ELISA) kit for Mouse LPS (F11123, Xi Tang, Shanghai, China). In short, serum samples were first diluted into 1:10 and added per well for 100 μl incubating 40 min at 37°C. The board was washed with washing solution 6 times before adding biotinylated antibody. Next, after enzyme conjugated with biotinylated antibody, 100 μl coloring solution and 100 μl stop solution per well were added subsequently. Microplate reader was used to measure the absorbance at 450 nm.

### Measurement of Hepatic Hydroxyproline Content

Total hepatic hydroxyproline levels were assayed to quantify liver collagen content using a commercial hydroxyproline assay kit (A030-2, Jiancheng, Nanjing, China). Briefly, 100 mg of wet liver samples was hydrolyzed for 20 min at 95°C. Next, samples were adjusted to neutrality and reacted with the corresponding detected resolution. The absorbance at 550 nm was recorded using microplate reader, and the concentration were calculated based on a standard product.

### Determination of Bile Acids

One hundred mg terminal ileum samples were mixed with NaOH and acetonitrile and centrifugated. After centrifugation, supernatants were placed in a chromatographic bottle for detection of bile acid levels (Majorbio Bio-Pharm Biotechnology, Shanghai, China). Chlorpropamide was used as an internal standard for bile acid levels. Bile acid concentrations of terminal ileum samples were qualitatively and quantitatively determined by LC-ESI-MS/MS analysis method (Waters Corp., Milford, MA). Mass spectrometry system included air curtain gas 40, ion spray voltage −4500 V, temperature 550°C, ion source GAS1:50, and ion source GAS2:50. Chromatographic separation was performed on a BEH C18 liquid chromatography column (100 × 2.1 mm, 1.8 μm, Waters Corp.) The injection quantity is 5 μl and mobile phase included phase A (0.1% formic acid in water) and phase B (0.1% formic acid in acetonitrile). Bile acids standards were used to identify different bile acids metabolites detected by LC-MS. Finally, the mass spectral peak area of the sample analyte was substituted into linear equation to calculate the concentration of bile acids levels.

### Stool Sample Collection, Microbial DNA Extraction and Sequencing

Fresh fecal samples were collected from the 24 mice on the day of sacrifice and immediately frozen at −80°C. DNA extraction and purification were performed with the QIAamp DNA Stool Minikit (Qiagen Ltd., Strasse, Germany) according to the manufacturer’s instructions. Briefly, weighing 150 mg wet stool were added to microcentrifuge tube containing lysis buffer and 25 µl Proteinase K, and vortexed to begin homogenization. Buffer AL (200 µl) was added to the sample and mixed. The QIAamp spin column labeled 1.5 ml microcentrifuge tube was placed for collecting DNA extraction. The quality of the extracted DNA was visualized on a 1% agarose gel. The V3-V4 hypervariable regions of the 16S rRNA genes were amplified using primers 338F (5′-ACT​CCT​ACG​GGA​GGC​AGC​AG-3′) and 806R (5′-GGACTACHVGGGTWTCTAAT-3′). Paired end sequencing was performed on the Illumina MiSeq PE300 platform (Illumina, San Diego, CA, United States) using standard protocols by Majorbio Bio-Pharm Technology Co. Ltd. (shanghai, China).

### Bioinformatic Analysis

The original data pre-processing and downstream bioinformatic analysis were primary conducted using Quantitative Insights into Microbial Ecology 2 (QIIME2, version 2021.04). Unless otherwise stated, default parameters for all programs and analyzes were used. Briefly, adapter sequences were trimmed with the script “qiime cutadapt trim-paired”. The DADA2 pipeline (with parameters “–p-trunc-len-f 269 –p-trunc-len-r 204”) was used for sequence filtering, dereplication, chimera identification and clustering high-quality reads into amplicon sequence variants (ASVs). The SILVA reference database (version 138) was used to annotate the ASVs (Quast et al., 2013). To correct for bias derived from sampling depth, subsampling was performed to 23,680 reads per biological sample. The alpha and beta diversity were calculated with “qiime diversity core-metrics-phylogenetic”. PICRUSt (version 2.4.1) was applied to predict functional genes and pathways.

### Data Visualization

GraphPad 7.0 (GraphPad Software, San Diego, CA, United States) was used to visualize data for mice features, qRT-PCR, histological data and bile acid contents. The gut microbiome analysis was visualized using Origin Pro 2021 (OriginLab, Northampton, MA, United States).

### Statistical Analysis

Statistical analyze were performed in GraphPad Prism 7.0. Comparisons made between multiple treatment groups were conducted with one-way analysis of variance (ANOVA) or Kruskal-Wallis test. Principal coordinate analysis (PCoA) was performed using the Unweighted Unifrac method. The linear discriminant analysis (LDA) effect size (LEfSe) method was implemented using LEfSe v1.0. ASV clustering was performed using the between-groups linkage method, and Pearson correlation calculation was conducted with SPSS Statistics 26 (SPSS Inc., Chicago, IL, United States). Correlation analysis of gut microbiome with bile acids in the terminal ileum was conducted using the nonparametric Spearman’s rank test with Origin Pro 2021. *p* < 0.05 was considered statistically significant.

## Results

### Rifaximin Alleviates Steatohepatitis in MCD Diet-Induced NASH Mice

**FIGURE 1:**
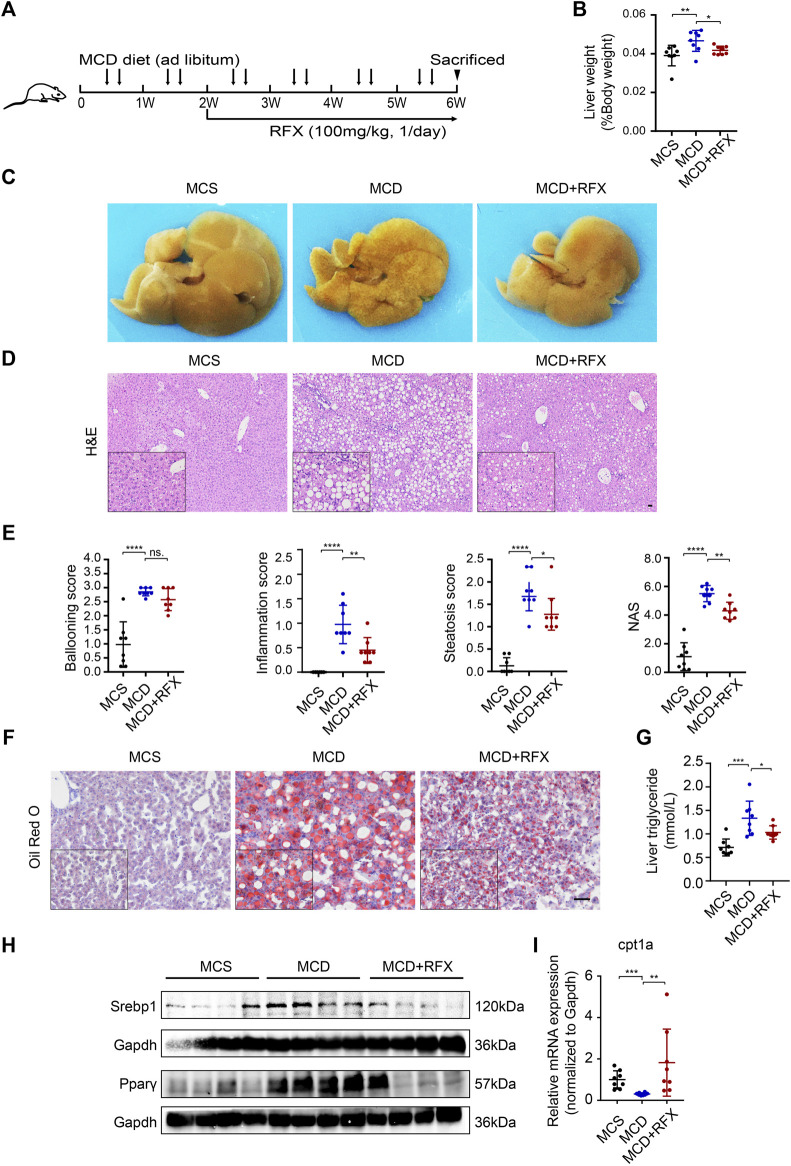
Administration of rifaximin ameliorates experimental non-alcoholic steatohepatitis (NASH) in MCD-fed mice. **(A)** Schematic illustration of the experimental design. **(B)** Liver to body weight ratio of C57BL/6 mice. **(C)** Representative macroscopic photographs of livers for mice fed with control diet (MCS) or MCD and with rifaximin gavage. **(D)** Representative images of paraffin-embedded liver sections stained with hematoxylin and eosin (H&E). Scale bars = 200 µm. **(E)** Histological scores of hepatic ballooning, inflammation, steatosis and total NAS based on H&E staining. **(F)** Representative histological staining for neutral lipid (Oil Red O) in mouse livers. Scale bars = 100 µm. **(G)** Hepatic triglyceride levels in mice. **(H)** Srebp1 and Pparγ expression in mouse liver. **(I)** Cpt1a mRNA levels in mouse livers. Gapdh is for normalization. Data are expressed as mean ± standard deviation. For B, E, G and I: *p < 0.05, **p < 0.01, ***p < 0.001, ****p < 0.0001 (*p* value was determined by one-way ANOVA). n = 8 mice per group.

To evaluate the effect of Rifaximin on non-alcoholic steatohepatitis (NASH) in mice, C57BL/6 mice were fed with a methionine- and choline-deficient (MCD) diet for 6 weeks to induce NASH. Rifaximin treatment was started after 2 weeks of the MCD diet and continued for 4 weeks (Figure 1A). As expected, MCD-fed mice had a significant decrease in body weight and increase in liver/body weight ratio compared with the control methionine and choline-sufficient (MCS)-fed mice (Figure 1B, Supplementary Figure S1A). Interestingly, rifaximin markedly reduced the liver/body weight ratio but did not alter body weight in MCD-induced NASH mice (Figure 1B, Supplementary Figure S1A). Macroscopic observation showed much more severe hepatic lipid deposition in MCD-fed mice compared with MCS-fed mice, whereas rifaximin treatment reduced liver lipid deposition in MCD-fed mice (Figure 1C). The Non-alcoholic Fatty Liver Disease Activity Score (NAS) incorporates hepatocellular ballooning, interlobular inflammation and steatosis scores, and has been used to evaluate the severity of NASH (Puri and Sanyal 2012). Rifaximin treatment decreased the interlobular inflammation and steatosis scores and NAS in MCD-fed mice. The ballooning score was numerically improved but with no significant difference in rifaximin-treated mice compared with untreated MCD-fed mice (Figures 1D,E). Consistent with the steatosis scores, Oil Red O staining and hepatic triglyceride level confirmed that elevated lipid deposition in the liver of MCD-fed mice was reduced by rifaximin treatment (Figures 1F,G and Supplementary Figure S1C). Srebp1 and Pparγ, which are essential regulators of lipid synthesis (Lee et al., 2018; Romano et al., 2021), were increased in MCD-fed mice and downregulated by rifaximin treatment (Figure 1H). To evaluate the activation of Srebp1, we investigated the location of Srebp1 expression using IHC. The data showed that the levels of Srebp1 in nuclear and cytoplasm were increased in the liver of MCD-fed NASH mice and reduced by rifaximin treatment (Supplementary Figure S1D). We also detected the expression of lipogenic genes regulated by Srebp1 using real-time qPCR and found that rifaximin treatment significantly reduced the expression of acetyl-CoA carboxylase-1 (ACC) in the mouse liver (Supplementary Figure S1E). Moreover, fatty oxidation related genes like carnitine palmitoyl transferase 1A (Cpt1a) mRNA was decreased in MCD-diet mice and rifaximin treatment reversed its transcriptional expression, indicating that rifaximin may also contribute to increasing hepatic lipid oxidation (Figure 1I). The MCD diet also increased plasma levels of alanine aminotransferase (ALT) and aspartate aminotransferase (AST), which were suppressed by rifaximin treatment (Supplementary Figure S1B). These data demonstrate a preventive effect of rifaximin on MCD diet-induced NASH in mice.

### Rifaximin Treatment Attenuates MCD Diet-Induced Hepatic Inflammation and Fibrosis in NASH Mice

Previous studies have revealed that the MCD diet induces hepatic inflammation (Li et al., 2020; Zhang et al., 2018). Serum lipopolysaccharide (LPS) serves as a trigger of hepatic inflammation (Okubo et al., 2016); we therefore measured LPS levels. The MCD diet resulted in a significant increase in serum LPS content, which was reduced by rifaximin treatment (Supplementary Figure S2A). The elevation of serum LPS levels caused increased expression of proinflammatory cytokines (Peng et al., 2021). Consistent with these results, qRT-PCR revealed increased expression of proinflammatory cytokine genes, including Tnfα and Mcp1 in the liver of the NASH mice (Supplementary Figure S2B). Expression of Tnfα was decreased significantly after rifaximin treatment, indicating that rifaximin attenuated the lobular inflammation of MCD-fed mice (Supplementary Figure S2B).

**FIGURE 2:**
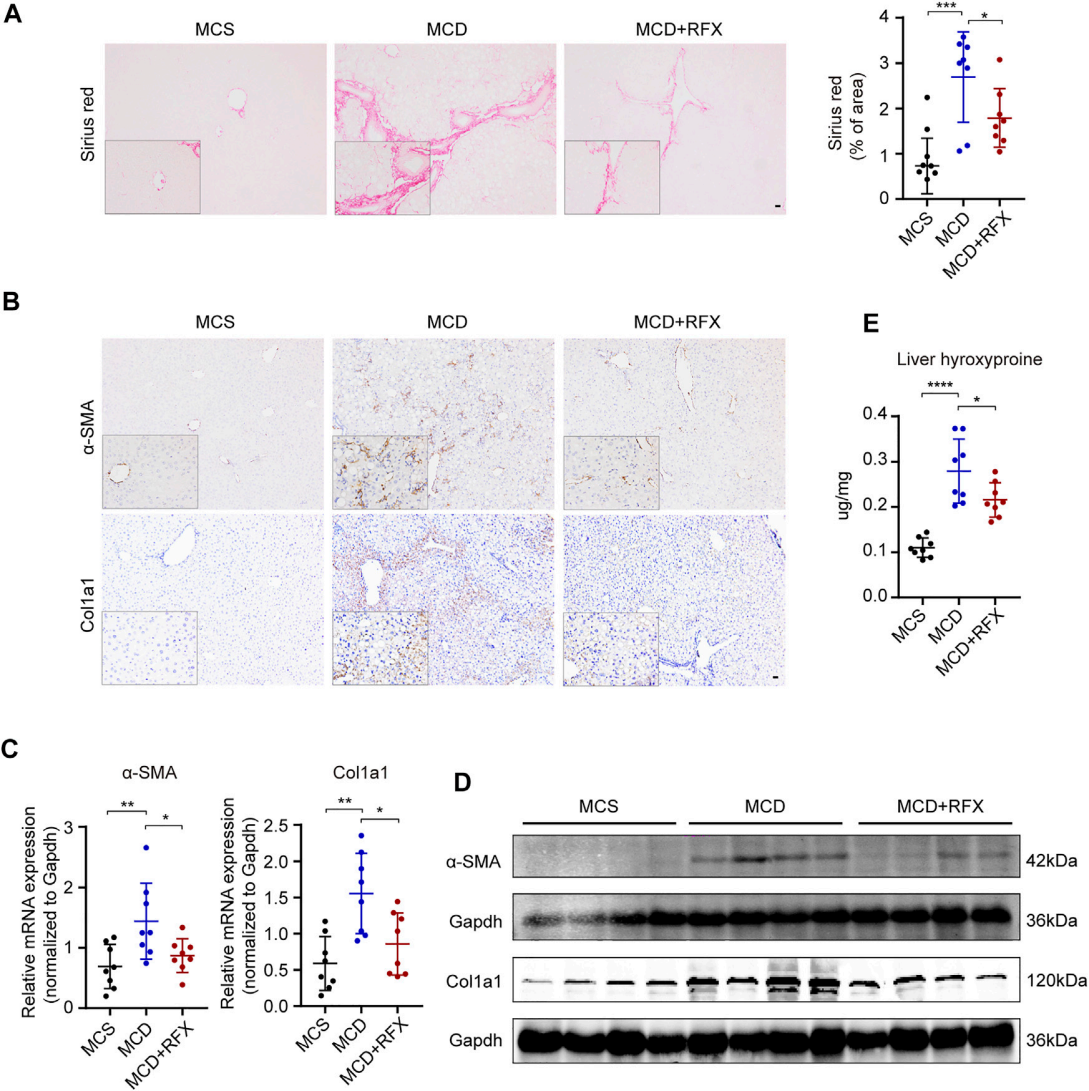
Rifaximin treatment alleviates MCD diet-induced hepatic fibrosis in mice. **(A)** Representative images of paraffin-embedded liver sections stained with Sirius Red (left panel) and semiquantitative analysis of Sirius Red staining positive area in mouse liver tissues (right panel). Scale bars = 200 µm. **(B)** Representative images of α-SMA and Col1a1 immunostaining. Scale bars = 200 µm. **(C)** qRT-PCR analysis of Acta2 and Col1a1 mRNA levels in mouse livers. Gapdh is for normalization. **(D)** Representative western blot analysis of α-SMA and Col1a1 in mouse livers. **(E)** Hydroxyproline content in mouse livers. Data are expressed as mean ± standard deviation. For A, C, E: *p < 0.05, **p < 0.01, ***p < 0.001, ****p < 0.0001 (*p* value was determined by one-way ANOVA). n = 8 mice per group.

Liver fibrosis is one of the most essential determinants to assess the stage and progression of NASH (Puri and Sanyal 2012). We found that collagen deposition and expression of collagen alpha 1 (Col1a1) and alpha smooth muscle actin (α-SMA) were increased in the livers of MCD-fed mice (Figures 2A–D). Rifaximin treatment significantly decreased collagen deposition and the expression of Col1a1 and α-SMA (Figures 2A–D). Liver hydroxyproline content was also enhanced in MCD-fed mice compared with MCS-fed mice (Figure 2E). However, after rifaximin treatment, hydroxyproline content in the liver was markedly reduced. These data indicate that rifaximin alleviates hepatic inflammation and fibrosis in NASH mice.

### Rifaximin Administration Alters the gut Microbiome in NASH Mice

**FIGURE 3:**
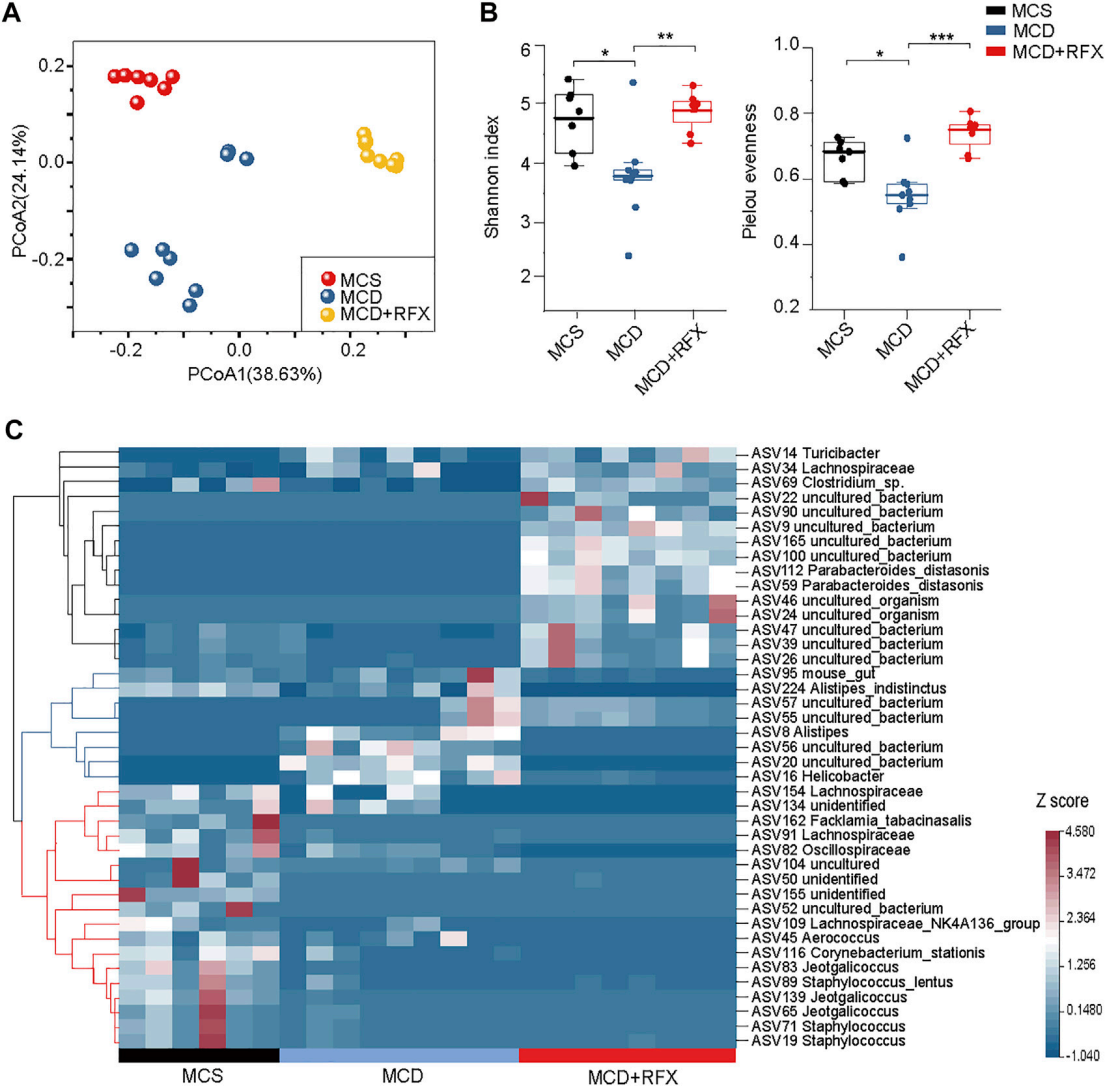
Effects of rifaximin on the gut microbiome in the MCD diet induced NASH mice. **(A)** Clear separation of samples by diets and rifaximin treatment was observed via Principal coordinate analysis (PCoA) of the Unweighted UniFrac distance based on ASVs. **(B)** Alpha diversity of three cohorts based on ASVs, measured in terms of the Shannon index and Pielou’s evenness. **(C)** The relative abundance of 41 differentially abundant ASVs identified by linear discriminant analysis effect size (LEfSe) (log(LDA) > 2 and *p* < 0.05) among the MCS diet, MCD diet, and rifaximin treatment groups through pairwise comparison between multiple groups. Clustering was performed using the Pearson measurement. *p < 0.05, **p < 0.01, ***p < 0.001.

As a non-systemic antibiotic, rifaximin targets the intestinal microbiome with minimal absorption. To evaluate the effect of rifaximin on the intestinal microbiome, we performed 16S rRNA amplicon sequencing of microbial DNA. Unweighted UniFrac principal coordinate analysis (PCoA) based on amplicon sequence variant (ASV) distributions revealed that the overall composition of the intestinal microbiome was significantly altered by the MCD diet (*p* = 0.001) and by rifaximin treatment (*p* = 0.001) (Figure 3A). The MCD diet significantly reduced alpha diversity indexes, such as Shannon diversity and Pielou’s evenness (Figure 3B). However, this effect was significantly reversed by rifaximin treatment.

A total of 41 ASVs were increased or decreased between samples, as identified by LEfSe analysis through pairwise comparison between multiple groups (log(LDA) > 2, *p* < 0.05) (Figure 3C). The 41 ASVs formed three main clusters when using the Pearson measurement (Figure 3C). A total of six ASVs including ASV162 (*Facklamia_tabacinasalis*) were enriched in the control diet group and decreased in the MCD diet-induced NASH mice. ASV8 (*Alistipes*), ASV16 (*Helicobacter*), ASV56 (*uncultured_bacterium*), and ASV20 (*uncultured_bacterium*) were significantly increased in the MCD-fed mice, but rifaximin treatment nearly depleted these ASVs. Moreover, rifaximin treatment increased abundant of 13 ASVs, including ASV69 (*Clostridium_sp.*) and ASV112 (*Parabacteroides_distasonis*) (Figure 3C). These results indicate that rifaximin further changes the MCD diet-induced alteration of the gut microbiome in NASH mice.

### Rifaximin Shifts the Metagenomic Function of the Intestinal Microbiome in the MCD Diet-Induced NASH Mice

**FIGURE 4:**
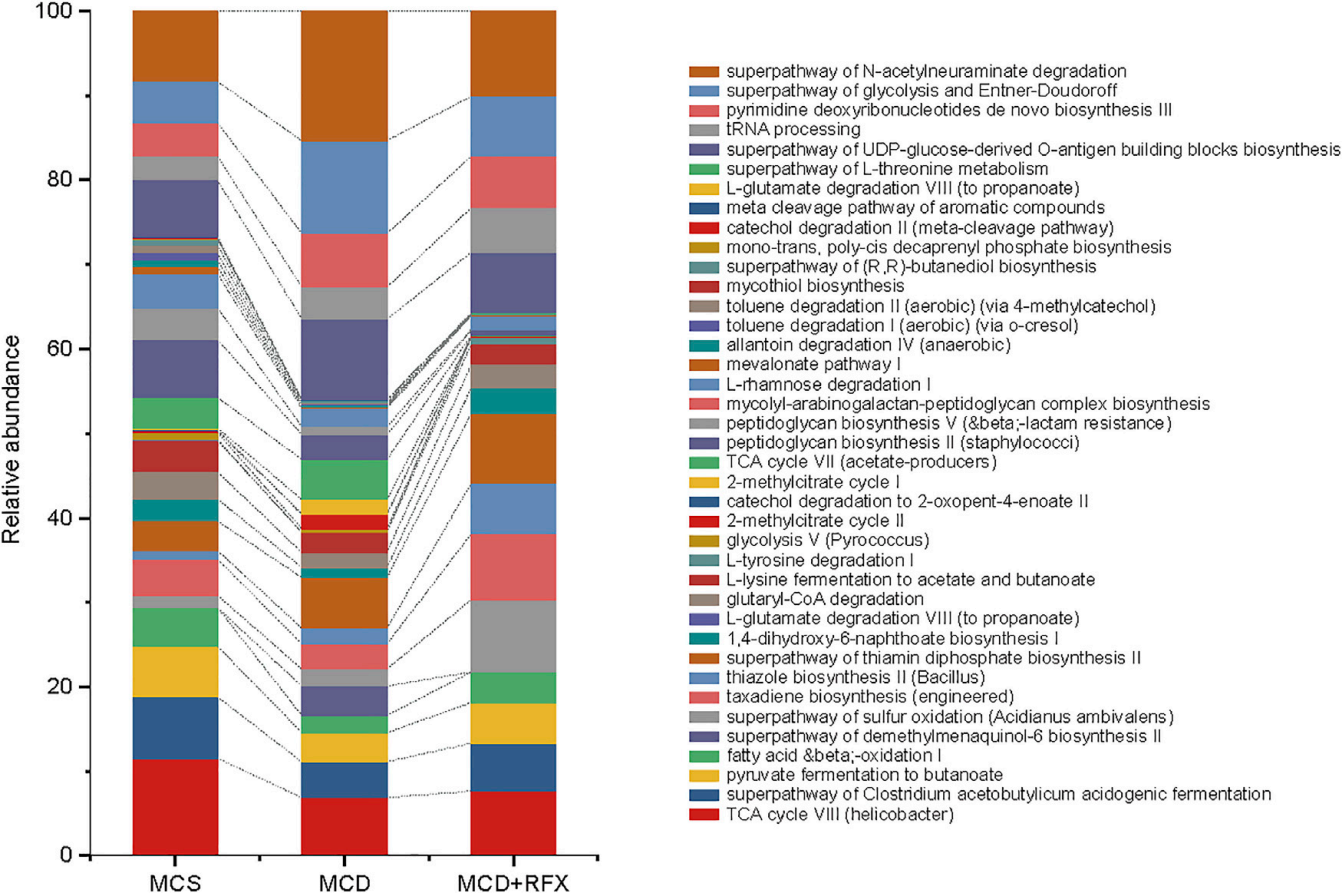
Functional pathways of the gut microbiome were altered by MCD diet and rifaximin treatment. KEGG pathways that were enriched or depleted in different treatment groups were identified by PICRUSt analysis and selected using linear discriminant analysis through pairwise comparison between multiple groups (log(LDA) > 2 and *p* < 0.05). Stacked bars display the relative abundance of different KEGG biological pathways.

The function of gut microbiome was predicted with PICRUSt (Langille et al., 2013). Biochemical pathways that were enriched or decreased by MCD diet and rifaximin treatment were assessed by LEfSe based on pathways from the Kyoto Encyclopedia of Genes and Genomes (KEGG) (Kanehisa et al., 2008). The MCD diet decreased four KEGG pathways: TCA cycle VIII (helicobacter), superpathway of Clostridium acetobutylicum acidogenic fermentation, pyruvate fermentation to butanoate, and fatty acid and beta oxidation I, whereas the superpathway of demethylmenaquinol-6 biosynthesis II was increased (Figure 4). Rifaximin administration enriched eleven KEGG pathways: superpathway of sulfur oxidation, taxadiene biosynthesis, thiazole biosynthesis II, superpathway of thiamin diphosphate biosynthesis II, 1, 4-dihydroxy-6-naphthoate biosynthesis I, L-glutamate degradation VIII, glutaryl-CoA degradation, L-lysine fermentation to acetate and butanoate, L-tyrosine degradation I, TCA cycle VIII, and fatty acid and beta oxidation I (Figure 4). Rifaximin decreased six KEGG pathways: which were related to the glycolysis V, 2-methylcitrate cycle II, catechol degradation to 2-oxopent-4-enoate II, 2-methylcitrate cycle I, TCA cycle VII (acetate-producers), and superpathway of demethylmenaquinol-6 biosynthesis II (Figure 4). Among these predicted pathways data using PICRUSt, we found rifaximin treatment altered microorganisms’ functional pathways in the MCD diet induced NASH mice, including fatty acid and beta oxidation I pathway et al. Therefore, we proposed that rifaximin treatment may be related to microorganisms’ lipid metabolism.

### Rifaximin Regulates the Level of DCA in the Ileum in the MCD Diet Induced NASH Mice

**FIGURE 5:**
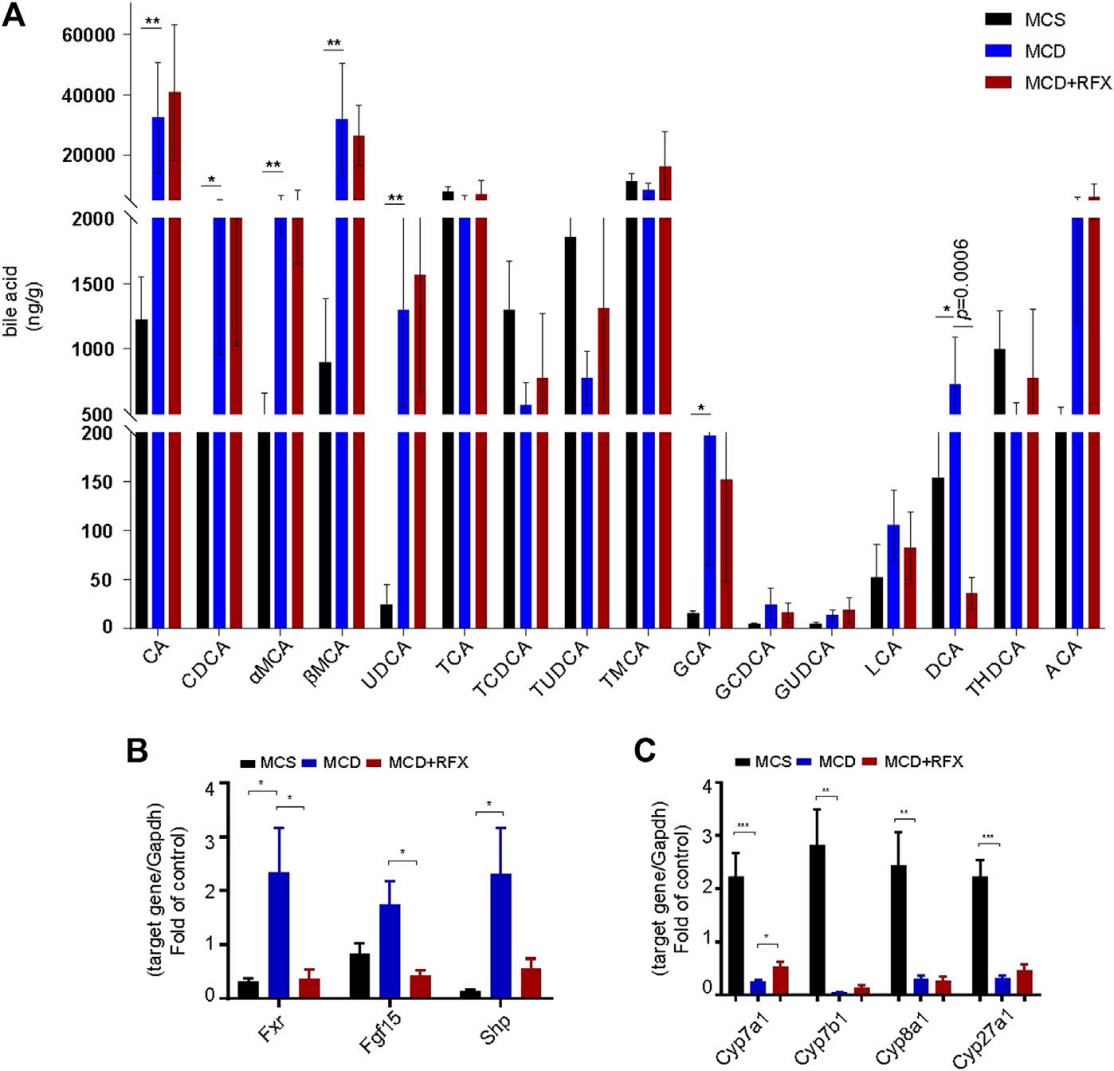
Impact of rifaximin on bile acid levels in the ileum and on the FXR signaling pathway. **(A)** Bile acid levels in the distal ileum. Levels of DCA decreased significantly after rifaximin treatment in MCD-fed mice n = 7 mice in each group. **(B)** Relative expression of Fxr and its target genes Fgf15 and Shp in the mouse intestine. **(C)** Relative expression of Cyp7a1, Cyp7b1, Cyp8b1, and Cyp27a1 mRNAs. Gapdh is for normalization. *p < 0.05, **p < 0.01, ***p < 0.001 (Kruskal-Wallis test). n = 8 mice in each group.

Disorders in the intestinal bile acids profile are associated with the development of Non-alcoholic fatty liver disease (NAFLD) (Hu et al., 2020). In this study, we investigated metabolic profiles of bile acids in the mouse ileum. The MCD diet induced notable upregulation of primary bile acids including cholic acid (CA), chenodeoxycholic acid (CDCA), muricholic acids (MCAs), ursodeoxycholic acid (UDCA), and glycine cholic acid (GCA) in mice. This suggested that the MCD diet may alter synthesis of primary bile acids in the liver (Figure 5A). Furthermore, the secondary bile acid deoxycholic acid (DCA) was also substantially higher in the MCD diet induced NASH mice (Figure 5A). Notably, rifaximin administration significantly suppressed the DCA content while the other bile acid levels remained statistically unchanged (Figure 5A). Because DCA is a natural agonist of farnesoid X receptor, we performed qRT-PCR to measure expression of genes related to Fxr-Fgf15 signaling in the intestine and its negative feedback loop in the liver (Makishima et al., 1999). Consistent with the increase in DCA levels associated with the MCD diet, *Fxr*, *Shp1,* and *Fgf15* were upregulated in the intestine while *Cyp7a1*, *Cyp7b1*, *Cyp8a1* and *Cyp27a1* were significantly downregulated in the liver (Figures 5B,C). Moreover, rifaximin reversed the activation of Fxr-Fgf15 signaling in the ileum and promoted expression of *Cyp7a1* in the liver (Figures 5B,C). These data suggest that rifaximin may attenuate MCD-induced NASH by modulating the DCA profile and downstream signaling in the intestine.

**FIGURE 6:**
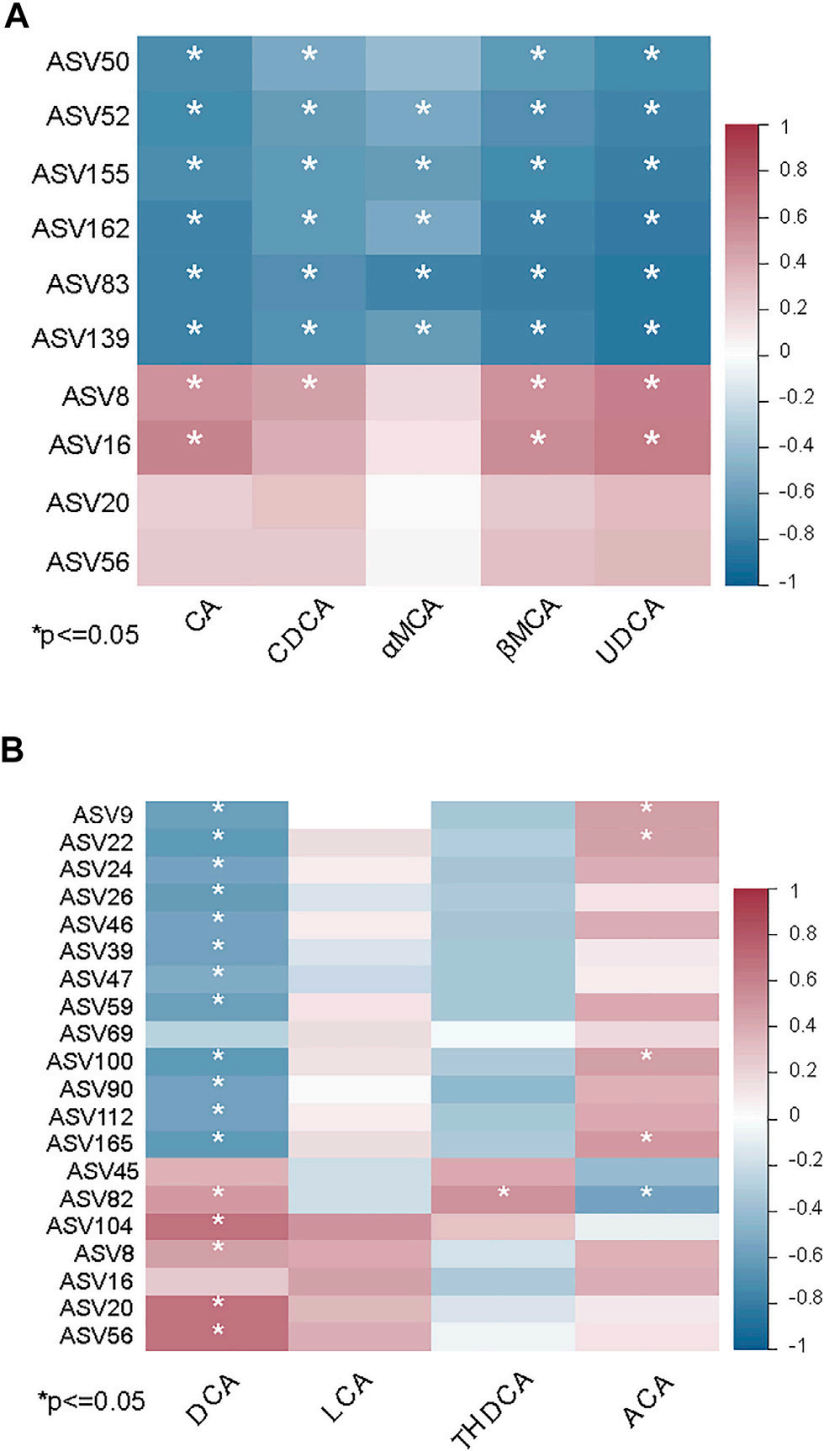
Associations between the gut microbiome and bile acid levels in MCD diet induced NASH mice. **(A)** Heatmap showing the correlation between primary bile acids and differentially abundant ASVs in MCS and MCD-fed mice. **(B)** Heatmap showing the correlation between secondary bile acids and differentially abundant ASVs in MCD-fed mice with and without rifaximin treatment. Asterisk refers to the significant negative or positive correlation. *p < 0.05 (Spearman’s correlation analysis). The r value is represented by gradient colors. n = 7 mice in each group.

Many studies have revealed that the gut microbiome has an impact on the synthesis of bile acids (Sayin et al., 2013; Wahlstrom, et al., 2016). To further identify interactions between the gut microbiome and levels of bile acids in the ileum, Spearman correlation analysis was performed to determine if there was an association between differentially abundant ASVs and bile acid levels. Eight ASVs were associated with the significant increase of primary bile acids in the MCD-fed mice compared with MCS-fed mice. The primary bile acids in mice, namely CA, CDCA, MCAs, UDCA, and GCA, were negatively correlated with six ASVs and positively correlated with two ASVs (Figure 6A). In contrast, DCA showed a negative correlation with rifaximin-elevated ASVs (belonging to *uncultured Desulfovibrionaceae, Mucispirillum, Rikenellaceae_RC9_gut_group, Dubosiella, Parabacteroides, Coriobacteriaceae_UCG-002, Muribaculaceae*) (Figure 6B). Notably, ASV8, ASV20 and ASV56 (belonging to the groups *Alistipes, Muribaculaceae, Bilophila,* respectively), which were enriched by the MCD diet but decreased by rifaximin treatment, exhibited a positive correlation with DCA (Figure 6B). These data indicate that rifaximin may decrease the DCA content by altering the gut microbiome in NASH mice.

## Discussion

To our knowledge, there has been no prior report investigating the preventive effect of rifaximin for NASH mice. There are three published studies that evaluate the efficacy of rifaximin in human NASH therapy, but with contradictory conclusions (Abdel-Razik, et al., 2018; Cobbold, et al., 2018; Gangarapu, et al., 2015). The discrepancies may be due to differing treatment parameters (dosage and duration). For example, in the open-label pilot study that revealed no therapeutic benefit of rifaximin for NASH patients, the rifaximin dosage was relatively low (800 mg/day) and the duration was relatively short (6 weeks) (Cobbold, et al., 2018). In this study, we found that rifaximin delivery markedly inhibited MCD diet-induced liver steatosis and deposition, and reduced NAS in mice. Thus, we speculate that rifaximin may exert beneficial effects on NASH at a more standard dosage (1,100 or 1,200 mg/day). Interestingly, Cheng et al. reported that low dose (1 mg/kg) rifaximin treated to the PXR-humanized healthy mice for 6 months significantly up-regulated triglyceride synthesis genes in liver. However, feeding diet and rifaximin dosage may all have impact on the hepatic lipid metabolism. Moreover, the relation between PXR and rifaximin in NASH requires further investigation.

Many clinical trials have shown that rifaximin can decrease serum LPS and its downstream proinflammatory cytokines (Abdel-Razik, et al., 2018; Gangarapu, et al., 2015). In the present study, we also found that rifaximin decreased hepatic inflammation, serum LPS, and Tnfα and Mcp1 expression in MCD-fed mice. Additionally, the effect of rifaximin in inhibiting NASH-fibrosis has been reported in a previous study in rats (Fujinaga, et al., 2020). Consistent with those prior results, we found that rifaximin ameliorated MCD diet-induced fibrosis in the NASH mouse liver. Consequently, we inferred that the reduction of hepatic inflammation may contribute to relief of hepatic fibrosis in NASH mice.

Rifaximin suppresses the activity of pathogens such as enterotoxigenic *Escherichia coli* and *Shigella* by altering microbial virulence (DuPont, 2011). One study showed that the clinical improvement of patients with ulcerative colitis or irritable bowel syndrome undergoing rifaximin treatment was associated with an increase in *Faecalibacterium* abundance (Ponziani et al., 2020). In a visceral hyperalgesia rat model, rifaximin reduced the overall small bowel bacterial burden but increased the abundance of *Lactobacillus* species (Xu et al., 2014; Bloom et al., 2021). These results implied that rifaximin treatment results in alteration of gut bacterial composition. In this study, rifaximin was also found to have a great capacity to regulate the gut microbial composition. Rifaximin treatment decreased the abundance of seven ASVs, such as ASV8 (*Alistipes*) and ASV16 (*Helicobacter*) while increasing several ASVs such as ASV112 (*Parabacteroides_distasonis*). Additionally, we determined that rifaximin enriched or depleted cohorts of new uncultured bacteria. The function and the impact of these new uncultured bacteria on humans was required to be defined. These data suggest that the effect of rifaximin on MCD diet-induced NASH may be partly attributed to modulation of the gut microbiome.

Studies have revealed that intestinal flora are closely associated with bile acid metabolism (Hutka et al., 2021). Intestinal bacteria convert primary bile acids into secondary bile acids by microbial biotransformation, including deconjugation and oxidation of hydroxyl groups (Wahlstrom, et al., 2016). Previous studies had identified *Clostridium* and *Eubacterium* belonging to the *Firmicutes* phylum are associated with secondary bile acids (Kitahara et al., 2001). In the present study, we investigated the ileal bile acid profiles and discovered that DCA was significantly decreased by rifaximin treatment. We also found that DCA levels were negatively correlated with several ASVs belonging to the *Firmicutes* phylum, including ASV26, ASV39, ASV47, which were increased after rifaximin treatment. Bile acids regulate metabolism in the host mainly through the bile acid receptor Fxr (Wahlstrom, et al., 2016). As a natural intestinal Fxr agonist, DCA activates the intestinal Fxr-Fgf15 signaling pathway to inhibit hepatic expression of *Cyp7a1* and *Cyp7b1,* which regulate lipid metabolism (de Aguiar Vallim et al., 2013; Kang et al., 2021). Our data show that rifaximin treatment significantly downregulated *Fxr* and *Fgf15* in the terminal ileum while upregulating *Cyp7a1* and *Cyp7b1* in the liver. Based on these results, we inferred that rifaximin may alter the gut microbiome and reduce DCA in the terminal ileum to attenuate MCD diet-induced NASH in mice. However, additional studies are needed to confirm this conclusion and explore the exact mechanism. Taken together, we found that rifaximin treatment relieved the MCD-induced NASH by modulating gut microbiome and decreasing DCA-Fxr signaling in the ileum. This study may serve as the basis for the development of promising treatment of NAFLD by administrating rifaximin in the future.

## Data Availability

The datasets presented in this study can be found in online repositories. The names of the repository/repositories and accession number(s) can be found below: https://www.ncbi.nlm.nih.gov/, PRJNA771219.
