# Supplementary material for: Rifaximin Ameliorates Non-alcoholic Steatohepatitis in Mice Through Regulating gut Microbiome-Related Bile Acids
Source: Front Pharmacol. 2022 Apr 4;13:841132. doi: 10.3389/fphar.2022.841132 (PMC9017645; doi:10.3389/fphar.2022.841132)
Supplement: Supplementary file 1 [file DataSheet1.docx]

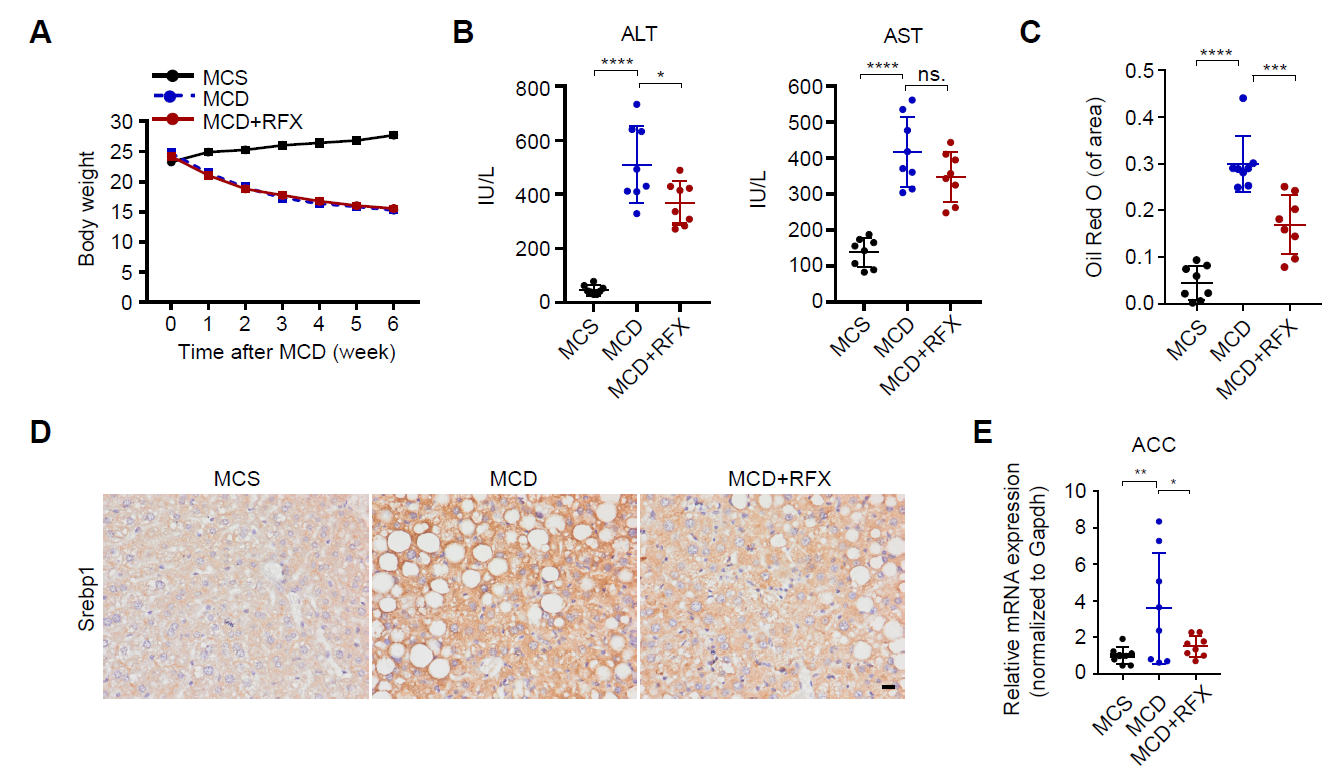


**Figure S1**|**(A)** Average total mouse body weight over time for each treatment group. **(B)** Serum alanine aminotransferase (ALT) and aspartate aminotransferase (AST) levels in mice. **(C)** Percentage of stained neutral lipid in mouse livers. **(D)** Representative images of Srebp1 immunostaining. Scale bars = 50 µm. **(E)** ACC mRNA levels in mouse livers. Gapdh is for normalization. *p < 0.05, **p < 0.01, ***p < 0.001, ****p < 0.0001 (P value was determined by one-way ANOVA). n = 8 mice in each group.


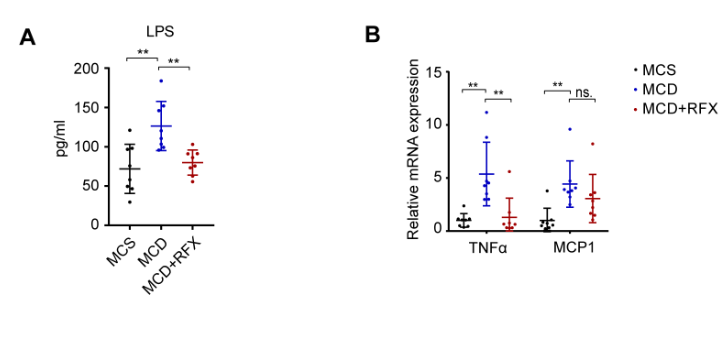


**Figure S2**|**(A)** Serum LPS levels of mice in the three treatment groups. **(B)** Relative expression of Tnfα and Mcp1 in the liver. Gapdh is for normalization. *p < 0.05, **p < 0.01, ***p < 0.001, ****p < 0.0001(P value was determined by one-way ANOVA). n = 8 mice in each group.

**Supplementary Table 1|**Primer sequences for qRT-PCR.

| Gene Symbol | Forward Primer & Reverse Primer |
| --- | --- |
| Acta2 | Forward: 5’- CTGTCCCTCTATGCCTCTGG -3’  Reverse: 5’- AGGGCTGTGATCTCCTTCTG -3’ |
| Col1A1 | Forward: 5’- TAAAGGGTCATCGTGGCTTC -3’  Reverse: 5’- GACGGCTGAGTAGGGAACAC-3’ |
| Cyp7a1 | Forward: 5’- TCATTGCTTCAGGGCTCCTG -3’  Reverse: 5’- TGGGCATCTCAAGCAAACAC -3’ |
| Cyp7b1 | Forward: 5’- TAGGCATGACGATCCTGAAA -3’  Reverse: 5’- TCTCTGGTGAAGTGGACTGAAA -3’ |
| Cyp8b1 | Forward: 5’- GATCCGTCGCGGAGATAAGG -3’  Reverse: 5’- CGGGTTGAGGAACCGATCAT -3’ |
| Cyp27a1 | Forward: 5’- TCTGGCTACCTGCACTTCCT -3’  Reverse: 5’- GTGTGTTGGATGTCGTGTCC -3’ |
| Fxr | Forward: 5’- TGGGCTCCGAATCCTCTTAGA -3’  Reverse: 5’- TGGTCCTCAAATAAGATCCTTGG -3’ |
| Fgf15 | Forward: 5’- GCCATCAAGGACGTCAGCA -3’  Reverse: 5’- CTTCCTCCGAGTAGCGAATCAG -3’ |
| Shp | Forward: 5’- TCTGCAGGTCGTCCGACTATTC -3’  Reverse: 5’- AGGCAGTGGCTGTGAGATGC -3’ |

**Supplementary Table 2|**Antibodies used for western blot.

| **Antibody** | **Company** | **Dilution** | **Catalogue number** |
| --- | --- | --- | --- |
| SREBP1 | Abcam | 1:500 | Ab28481 |
| PPARγ | Santa Cruz Biotechnology | 1:500 | Sc-7273 |
| α-SMA | Abcam | 1:500 | ab5694 |
| COL1A1 | Boster | 1:1000 | BA0325 |
| GAPDH | Bioworld | 1:5000 | AP0063 |
